# Work ability and anthropometric indices correlate with cardiovascular risk factors in public sector employees: Cross‐sectional study

**DOI:** 10.1002/hsr2.1728

**Published:** 2023-11-21

**Authors:** Angela Arthur, Anthony Mensah‐Asamoah, Eleazer Kofi Mensah Brown, Anita Kabuki Ocansey, Prince De‐Gaulle Deku, Monday Omoniyi Moses

**Affiliations:** ^1^ Department of Physiotherapy and Sport Science, Faculty of Allied Health Sciences, College of Health Sciences Kwame Nkrumah University of Science and Technology Kumasi Ghana

**Keywords:** blood pressure, body mass index, public sector employees, weight to hip ratio, work ability

## Abstract

**Background and Aims:**

Understanding the correlation of work ability (WA) and anthropometric indices with cardiovascular diseases (CVDs) risk factors among public sector employees (PSE) is vital for policy direction. This study examined the correlation between work ability, anthropometric indices, and cardiovascular risk factors among PSEs.

**Methods:**

The cross‐sectional study had 254 (mean age = 37.18 ± 10.34) PSE. A self‐reported WA index was used to measure WA. Blood pressure (BP), body mass index (BMI), waist circumference (WC), hip circumference (HC), waist to hip ratio (WHR), and visceral fat were measured. Lifestyle CVDs risk history was also obtained.

**Results:**

3.9% had moderate, 51.2% good, and 44.9% excellent WA. 37.4% overweight, 20.1% obese, 19.7% hypertension history, 67.7% no physical activity history. WA correlates with increased systolic BP, BMI, WC, WHR, weight to height ratio, and visceral fat significantly. Age 24−29 (aOR = 26.38), 30‐39 (aOR = 7.52), and 40‐49 (aOR = 4.94) independently predict excellent WA. Overweight (aOR = 0.44) independently predict decreased excellent WA.

**Conclusion:**

Participants were hypertension‐prone, had increased WC, WHR, physically inactive, overweight, and obese. WA and anthropometric indices of the participants predict CVDs risks. Workplace health care strategy should be put in place to control BP, BMI, WC, WHR, weight to height ratio, and visceral fat as CVDs risk factors.

## 1 INTRODUCTION

Work capacity/ability (WA) relies on composite factors such as physical and mental health, skills, competences, motivation, attitudes, work environment, and organizational supports.^1^, ^2^ WA also has direct influences on productivity, quality, contentment, well‐being, and employability for companies and workers.^1^ Study has reported a correlation between job capacity limitations and several factors such as musculoskeletal discomfort, chronic illness, absenteeism, premature retirement, and overall mortality.^3^ Overweight or obesity employees are often subject to negative stereotypes, such as being seen as lacking in motivation, laziness, and lower levels of competence compared to employees who have a normal weight.^4^ Reduced WA may indicate cardiovascular diseases (CVDs), physical, mental, and social instability.^5^, ^6^ Physical, mental, and social instability can lead to negative weight, body mass index (BMI), waist circumference (WC), and waist‐to‐hip ratio, obesity, and related health hazards.^7^, ^8^ Predicting cardiovascular risk factors in working populations, particularly in the public sector, has grown in popularity.^9^

Studies have shown that public sector employees (PSE) work under pressure, are overworked, and spend much time working tediously, neglecting their health.^10^, ^11^ In Ghana, most PSE are expose to stress workload, role ambiguity, role insufficiency, work family relations, adverse working conditions, career development, time pressure, individual factors, organizational adjustment, changing global scene, and working under two perceived supervisors.^12^, ^13^ The nature of the work of PSE is such that they may not even have spare time to eat well, engage in physical activities, or even go for regular medical checkups.^14^, ^15^ PSE who may be prone to CVDs are detected and diagnosed late, hence there is low survival rate most times.^16^ Optimal performance at work will only be possible when cardiovascular risk factors such as hypertension, overweight, obesity, physical inactivity, diabetes, alcohol consumption, smoking status, and poor eating habits^17^, ^18^, ^19^ are at their lowest level in relation to workload.^20^ Anthropometric indices, such as absolute total fat and adipose tissue distribution, have been demonstrated in previous research to have a strong correlation with the chances of developing diabetes, hypertension, hyperlipidemia, and CVD.^21^, ^22^, ^23^ Other studies have also found total body fat, or BMI, rather than its distribution, is the stronger predictor of metabolic risks.^24^, ^25^ Researchers have further proven that increased work demands, and workplace exposures may contribute to the onset of CVDs and are linked to future sick days and early retirement.^26^, ^27^, ^28^

Ghana, a developing nation with limited resources, continues to experience an increase in the CVDs mortality rate, which accounts for approximately 50% of fatalities.^29^, ^30^ Workplace CVD prevention would require understanding how job abilities and anthropometric indices predict cardiovascular risk factors. CVD are among the leading causes of death worldwide with negative effects on human productivity.^31^, ^32^ Further information on the interaction of WA and anthropometric indices with CVDs risk factors among PSE is vital for policy direction. This study examined the relationship between WA, anthropometric indices, and cardiovascular risk factors among PSE. This study presents evidence on WA and anthropometric indices as predictors of CVDs risk factors in PSE, which will influence future research and workplace interventions to promote employee health and well‐being.

## 2 METHODS

A cross‐sectional study design was employed in this study conducted among employees at public institutions within the Kumasi Metropolitan Assembly (KMA). KMA is one of the 260 metropolitan, municipal, and district assemblies in Ghana. KMA is one of the 43 districts in Ashanti Region in Ghana, with Kumasi as its administrative capital. The study was conducted in line with the STrengthening the Reporting of Observational studies in Epidemiology (STROBE) for observational studies.^33^

The study participants were recruited through a random sampling technique. The random sampling technique ensured that each participant has equal opportunity to be selected as established the literature.^34^ The sample size was obtained by the formula^35^: *n* = (Z^2^ x PQ)/e^2^, where: Z is the standard normal variate at a confidence interval of 95% = 1.96. *p* is the prevalence = 7% of CVD in Ghana, e is the margin of error = 0.05, and Q = 1−*p*. n (minimum number of participants) = 1.96^2^ x 0.07 x (1−0.07)/0.05^2^ = 100. Hence, a minimum of 100 participants was required for the study.^35^ But to increase statistical power, 254 participants were recruited for the study. Eight (8) professional sections participated in the study with proportional representation as: Finance (22, 8.7%), Procurement (11, 4.3%), Human Resource/Administration (47,18.5%), Health Professionals(27, 10.6%), Education (25, 9.8%), Security (17, 6.7%), Sports Directorate (76, 29.9%), and Cleaners(29, 11.4%).

### 2.1 Inclusion and exclusion criteria

Full time employees of public institutions within the KMA were included in the study. The study used PSE who experience high level of bureaucratic practise, tend to avoid unpaid overtime work, whose remunerations are often disbursed early and rarely delayed, more likely to be absent from work, tend to experience less stringent oversight responsibilities, and have job security when compared to private sector employees who were excluded. Employees of private and part time employees of public sectors are habitually dedicated to their job, rarely go to work late and often endure any challenge encountered for the sake of not losing one's job.

### 2.2 Instrumentation

Demographic data on age, educational level, marital status, number of children, residence, religion, work experience (years), and working section was obtained. Lifestyle related information (e.g., smoking status, physical activity, and alcohol consumption) was also obtained. Omron body composition analyzer and nonelastic tape measure were used to measure anthropometric indices. An Omron sphygmomanometer was used to measure blood pressure (BP) and heart rate of the study participants.

The WA Index (WAI), a self‐assessment instrument, was administered to measure the work ability of the participants.^36^ The WAI describes how well an employee can do his/her job.^37^ It is recommended as a diagnostic instrument for the development of measures for health support and the identification of employees who require medical care.^38^, ^39^ WAI has been well used in academic and clinical settings, with a reliability value of 0.73.^40^, ^41^, ^42^ The WAI measures seven aspects of WA: current WA compared with the lifetime best; WA in relation to the demands of the job; number of current diseases diagnosed by physician; estimated work impairment due to diseases; sick leave during the past year (12 months); own prognosis of work ability 2 years from now; and mental capacities.^36^ The maximum score on the index is 49 points, and the minimum is 7. Except for items 2, 3, and 7, for which there are specific scoring criteria, the total score is calculated by aggregating the points of each item. For Item 2 (labor aptitude relative to job requirements). The labor aptitude score for the job's physical requirements is multiplied by 1.5 (answers between 3 and 5). Multiply by 0.5 the work aptitude score for the mental demands of the task. Answers between 1 and 2. The WA score for the physical demands of the job is multiplied by 0.5 (answers from 1 to 2).^36^ The WA score for the mental demands of the job is multiplied by 1.5 (answers from 3 to 5) for work that is both physically and mentally demanding the WA score remains unchanged. Details procedures and scoring of the WAI has been reported^43^, ^44^, ^45^, ^46^ Total score obtained is interpret based on Table 1.

**Table 1:** WAI Cumulative Index (Range 7−49 points).^25^

Points	Work ability	Objective of measures
7−27	Poor	Restore work ability
28−36	Moderate	Improve work ability
37−43	Good	Support work ability
44−49	Excellent	Maintain work ability

### 2.3 Anthropometric measurements

The height and weight were measured to the nearest 0.5 cm and 0.1 kg, respectively, while wearing light apparel and without shoes. The BMI formula was (weight [kg])/height^2^ (m). Data obtained were categorized according to standard.^47^, ^48^ The average of the waist and pelvic circumferences was used for further analysis. Hip circumference (HC) was measured at the level of the greater trochanters. The waist to hip ratio (WHR) was then computed.

### 2.4 CVD risk factor components

As CVD risk factor components, BP, heart rate, BMI, and visceral fat were measured and recorded. After each participant had sat for at least 10 min, two consecutive BP readings were taken from the participant's right arm. The systolic and diastolic BPs were recorded to the closest mmHg. If the difference between the two readings is greater than 4 mmHg, a third reading is taken. The subsequent analysis used the average of the two BP measurements. As the onset and disappearance of Korotkoff sounds, systolic and diastolic BP (SBP and DBP, respectively) were determined. Visceral adiposity was determined and recorded. The questionnaire on knowledge of cardiovascular risk factors (Q‐FARCS)^49^ was modified to elicit information on CVD risk lifestyles. The information elicited includes history of diabetes (No or Yes), history of smoking (Never, Former, and Current), alcohol (Never, Former, and Current), hypertension (No or Yes), physical activities participation (Never, Sometimes, Always), time of physical activity (Less than 1 h, 1−2 h, More than 2 h), type of physical activity aerobics, if any (Gym/walking/football), too much intake of salt (No or Yes), take fruits and vegetables (Never, Sometimes, Always), and experience of heart attack or pain (No or Yes) were obtained.

Before data collection, the participants were given a thorough explanation of the study protocol and assurances of anonymity. Also, ethical approval was obtained from the Committee on Human Research, Publication, and Ethics, School of Medical Sciences, Kwame Nkrumah University of Science and Technology (Ref: CHRPE/AP/024/22). Authors declare that the work has been done in accordance with the declaration of Helsinki statement of ethical principles for medical research involving human subjects, including research on identifiable human material and data.

#### 2.4.1 Statistical analysis

Microsoft Excel 2019 was used to enter, cleanse, and code the collected data. All statistical analyses were conducted using Statistical Package for the Social Sciences (SPSS) Version 26.0 and GraphPad Prism Version 8.0 (GraphPad Software, www.graphpad.com). Means and standard deviations served as the representations of parametric variables while medians and interquartile ranges served as those of nonparametric continuous variables. Variables of a categorical nature were represented as frequencies and percentages. Utilizing a Pearson *χ* ^2^ test statistic and logistic regression analysis, correlation of cardiovascular risk factors as independent variables and WA as dependent were determined. A confidence interval of 95% and a p‐value deemed statistically significant at the 0.01 and 0.05 levels (2‐tailed).

## 3 RESULTS

This section presents the findings from the study. Table 2 shows that majority of the PSE in the study were within the ages of 24−29 years (32.7%), 50.4% were males, and 59.8% were married. 36.2% had no child, 79.5% had tertiary education and 46.1% had worked for 1−5years while 94.1% were affiliated to Christian's religion. Out of the eight professional sections participated in the study with proportional representation as: Finance (22, 8.7%), Procurement (11, 4.3%), Human Resource/Administration (47, 18.5%), Health Professionals(27, 10.6%), Education (25, 9.8%), Security (17, 6.7%), Sports Directorate (76, 29.9%), and Cleaners (29, 11.4%).

**Table 2:** Demographic characteristics of study participants.

Variables		Frequency (*n* = 254)	Percentage (%)
Age Group (Years)	Age (π ± SD)	37.18 ( ± 10.343)	
	24−29	83	32.7
	30−39	77	30.3
	40−49	47	18.5
	50−59	47	18.5
Gender	Male	128	50.4
	Female	126	49.6
Marital status	Single	92	36.2
	Married	152	59.8
	Divorced/Widowed	10	3.9
Number of children	0	92	36.2
	1−2	67	26.4
	3−4	80	31.5
	5−6	15	5.9
Educational level	JHS	11	4.3
	SHS	41	16.1
	Tertiary	202	79.5
Work experience (years)	<1	20	7.9
	1−5	117	46.1
	6−10	31	12.2
	11−20	45	17.7
	>20	41	16.1
Residence	Rural	28	11
	Urban	226	89
Religion	Christian	239	94.1
	Muslim	15	5.9
Body mass index category	Underweight	4	1.6
	Normal	104	40.9
	Overweight	95	37.4
	Obese	51	20.1

According to Tables 3, 99.6% had no history of diabetes, 10.6% had history of smoking, and 89.4% had never taken alcohol. 57.5% were at least overweight, 38.6% had a history of hypertension, and 67.7% had never taken part in any physical activity.

**Table 3:** History of cardiovascular risk factors of the participants.

Variables		Frequency (*n* = 254)	Percentage (%)
History of diabetes	No	253	99.6
	Yes	1	0.4
History of smoking	Never	249	98
	Former	3	1.2
	Current	2	0.8
History of alcohol	Never	227	89.4
	Former	11	4.3
	Current	16	6.3
History of hypertension	No	156	61.4
	Yes	98	38.6
Physical activities	Never	172	67.7
	Sometimes	62	24.4
	Always	20	7.9
Duration of physical activity	Less than 1 h	58 of 82	70.7
	1−2 h	22 of 82	26.8
	More than 2 h	2 of 82	2.5
Type of physical activity	Aerobics	54 of 82	65.9
	Gym/walking/football	28 of 82	34.1
Too much intake of salt	No	251	98.8
	Yes	3	1.2
Take fruits and vegetables	Never	1	0.4
	Sometimes	222	87.4
	Always	31	12.2
Have experienced heart attack or pain	No	250	98.4
	Yes	4	1.6

**Figure 1:**
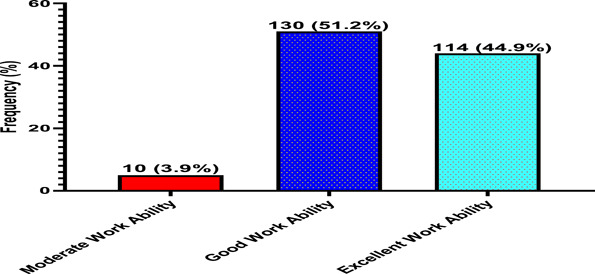
Proportion of work ability among study participants.

Figure 1 indicates that 51.2% had good work index while 44.9% had excellent WA. From Table 4, as the age of participants with excellent WA increased, their WA declined from 46.5% to 3.5%. Male participants with good WA are more than female (52.3% > 48.2%) while the case reversed with excellent WA (47.7% < 51.8%). Workers in the procurement department had higher WA than others (18.5%). Workers without children had better WA (36.2%). Participants who had worked for between 1 and 5 years had better WA (46.1%). Based on BMI category, the WA index of underweight participants was better (40.9%).

**Table 4:** Association of demographic information with work ability.

Variable	Moderate work ability (*n* = 10)	Good work ability (*n* = 130)	Excellent work ability (*n* = 114)	Grand total (*n* = 254)	*p* Value
Age group (years)	1 (10.0)	29 (22.3)	53 (46.5)	83 (32.7)	<0.001
	1 (10.0)	34 (26.2)	42 (36.8)	77 (30.3)	
	1 (10.0)	31 (23.8)	15 (13.2)	47 (18.5)	
	7 (70.0)	36 (27.7)	4 (3.5)	47 (18.5)	
Gender	5 (50.0)	68 (52.3)	55 (48.2)	128 (50.4)	0.818
	5 (50.0)	62 (47.7)	59 (51.8)	126 (49.6)	
Working section	1 (10.0)	18 (13.8)	14 (12.3)	33 (13.0)	0.432
	2 (20.0)	20 (15.4)	25 (21.9)	47 (18.5)	
	1 (10.0)	14 (10.8)	12 (10.5)	27 (10.6)	
	1 (10.0)	5 (3.9)	19(16.7)	25 (9.8)	
	1 (10.0)	11 (8.5)	5 (4.4)	17 (6.7)	
	3 (30.0)	41 (31.5)	32 (28.1)	76 (29.9)	
	1 (10.0)	21 (16.2)	7 (6.1)	29 (11.4)	
Marital status	1 (10.0)	40 (30.8)	51 (44.7)	92 (36.2)	**0.002**
	9 (90.0)	80 (61.5)	63 (55.3)	152 (59.8)	
	0 (0.0)	10 (7.7)	0 (0.0)	10 (3.9)	
Number of children	1 (10.0)	40 (30.8)	51 (44.7)	92 (36.2)	<**0.001**
	0 (0.0)	28 (21.5)	39 (34.2)	67 (26.4)	
	7 (70.0)	54 (41.5)	19 (16.7)	80 (31.5)	
	2 (20.0)	8 (6.2)	5 (4.4)	15 (5.9)	
Educational level	0 (0.0)	7 (5.4)	4 (3.5)	11 (4.3)	0.473
	0 (0.0)	20 (15.4)	21 (18.4)	41 (16.1)	
	10 (100.0)	103 (79.2)	89 (78.1)	202 (79.5)	
Work experience (years)	0 (0.0)	8 (6.2)	12 (10.5)	20 (7.9)	<**0.001**
	2 (20.0)	47 (36.2)	68 (59.6)	117 (46.1)	
	0 (0.0)	12 (9.2)	19 (16.7)	31 (12.2)	
	2 (20.0)	32 (24.6)	11 (9.6)	45 (17.7)	
	6 (60.0)	31 (23.8)	4 (3.5)	41 (16.1)	
Residence	1 (10.0)	15 (11.5)	12 (10.5)	28 (11.0)	0.963
	9 (90.0)	115 (88.5)	102 (89.5)	226 (89.0)	
Religion	9 (90.0)	126 (96.9)	104 (91.2)	239 (94.1)	0.145
	1 (10.0)	4 (3.1)	10 (8.8)	15 (5.9)	
Body mass index category	2 (20.0)	46 (35.4)	56 (49.1)	104 (40.9)	<**0.001**
	0 (0.0)	1 (0.8)	3 (2.6)	4 (1.6)	
	1 (10.0)	60 (46.2)	34 (29.8)	95 (37.4)	
	7 (70.0)	23 (17.7)	21 (18.4)	51 (20.1)	

*Note*: Bold values indicate factorial analysis outcomes.

According to Table 5, WA (cOR = 0.95, 95% CI = (0.93−0.97); *p* = < 0.0001) significantly increased systolic BP, weight (cOR = 0.98, 95% CI = (0.96−1.00); *p* = 0.041), and BMI (cOR = 0.94, 95% CI = (0.90−0.99); *p* = 0.027) by 5% and 10% respectively. Also, increased WC(cOR = 0.97, 95% CI = (0.95−0.99); *p* = 0.003), and WHR (cOR = 0.001, 95% CI = (0.00−0.08); *p* = 0.002) were significantly associated with 5%, 3%, 100% and 100% respectively of reduced WA. On the other hand, visceral fat (cOR = 0.88, 95% CI = (0.82−0.95); *p* = 0.002) were associated with decreased excellent WA.

**Table 5:** Association of work ability and components of cardiovascular risk factor.

Variable	Median (IQR)	*p* Value	cOR (95% CI)	*p* Value
Systolic blood pressure	122.00 (114.00−133.00)	0.0100	0.95 (0.93−0.97)	**0.001**
Diastolic blood pressure	79.00 (72.00−84.00)	0.0890	0.93 (0.91−0.96)	**0.001**
Heart rate	79.00 (74.00−86.00)	0.1450	0.98 (0.96−1.01)	0.201
Height	164.25 (159.00−171.08)	0.2380	1.00 (0.98−1.03)	0.859
Weight	71.60 (64.08−77.83)	0.0030	0.98 (0.96−1.00)	**0.041**
BMI	26.07 (23.07−29.26)	<0.0001	0.94 (0.90−0.990	**0.027**
Waist circumference	87.00 (80.00−95.00)	<0.0001	0.97 (0.95−0.99)	**0.003**
Hip circumference	102.0 0 (95.00−109.00)	0.0070	0.99 (0.97−1.01)	0.198
Weight to hip ratio	0.86 (0.81−0.89)	<0.0001	0.001 (0.00−0.08)	**0.002**
Weight to height ratio	0.53 (0.47−0.58)	<0.0001	0.01 (0.00−0.25)	**0.005**
Percentage body fat	31.30 (22.43−39.75)	0.0240	1.00 (0.97−1.02)	0.701
Muscle mass	29.90 (25.70−36.73)	0.0110	1.01 (0.97−1.05)	0.625
Visceral fat	7.00 (5.00−9.00)	<0.0001	0.88 (0.82−‐0.95)	**0.002**
Resting metabolic rate	1514.00 (1362.00−1690.25)	0.2770	1.00 (1.00−1.00)	0.143

*Note*: Bold values indicate factorial analysis outcomes.

From Table 6, most of the participants (99.6%) had no history of diabetes, 98.0% never smoked, 89.4% never drank alcohol, 80.3% had no history of hypertension, and 67.7% never engaged in any planned physical activity but had at least good work index. 70.7% of those who engaged in planned physical activity spent less than an hour per session participating in aerobic exercise (65.4%) and 34.6% with gym activity/walking/football. 98.8% do not take too much of salt. 87.4% consumed fruits and vegetables sometimes. According to the self‐reported information from the participants, 98.4% had never experienced heart attack/pain while only 1.6% experienced such. The 1.6% participants had good work ability index.

**Table 6:** Work ability and history of cardiovascular risk factors.

Variable	Moderate work ability (*n* = 10)	Good work ability (*n* = 130)	Excellent work ability (*n* = 114)	Total (*n* = 254)	*p* Value
*History of diabetes*					**0.000**
No	9 (90.0)	130 (100.0)	114 (100.0)	253 (99.6)	
Yes	1 (10.0)	0 (0.0)	0 (0.0)	1 (0.4)	
*History of smoking*					0.039
Never	9 (90.0)	126 (96.9)	114 (100.0)	249 (98.0)	
Former	1 (10.0)	2 (1.5)	0 (0.0)	3 (1.2)	
Current	0 (0.0)	2 (1.5)	0 (0.0)	2 (0.8)	
*History of alcohol consumption*					0.051
Never	8 (80.0)	112 (86.2)	107 (93.9)	227 (89.4)	
Former	0 (0.0)	6 (4.6)	5 (4.4)	11 (4.3)	
Current	2 (20.0)	12 (9.2)	2 (1.8)	16 (6.3)	
*History of hypertension*					**<0.000**
No	4 (40.0)	87 (66.9)	113 (99.1)	204 (80.3)	
Yes	6 (60.0)	43 (33.1)	1 (0.9)	50 (19.7)	
*Physical activities*					0.820
Never	7 (70.0)	84 (64.6)	81 (71.1)	172 (67.7)	
Sometimes	2 (20.0)	34 (26.2)	26 (22.8)	62 (24.4)	
Always	1 (10.0)	12 (9.2)	7 (6.1)	20 (7.9)	
*Time of physical activity*					0.407
Less than 1 h	3 (100.0)	29 (63.0)	26 (78.8)	58 (70.7)	
1−2 h	0 (0.0)	16 (34.8)	6 (18.2)	22 (26.8)	
More than 2 h	0 (0.0)	1 (2.2)	1 (3.0)	2 (2.4)	
*Type of physical activity*					0.214
Aerobics	1 (33.3)	28 (60.9)	24 (75.0)	53 (65.4)	
Gym/walking/football	2 (66.7)	18 (39.1)	8 (25.0)	28 (34.6)	
Too much intake of salt					0.235
No	10 (100.0)	127 (97.7)	114 (100.0)	251 (98.8)	
Yes	0 (0.0)	3 (2.3)	0 (0.0)	3 (1.2)	
*Take fruits and vegetables*					0.3080
Never	0 (0.0)	1 (0.8)	0 (0.0)	1 (0.4)	
Sometimes	8 (80.0)	109 (83.8)	105 (92.1)	222 (87.4)	
Always	2 (20.0)	20 (15.4)	9 (7.9)	31 (12.2)	
*Have experienced heart attack or pain*					0.144
No	10 (100.0)	126 (96.9)	114 (100.0)	250 (98.4)	
Yes	0 (0.0)	4 (3.1)	0 (0.0)	4 (1.6)	

*Note*: Bold values indicate factorial analysis outcomes.

From Table 6, there was significant association between history of diabetes (*p* < 0.000), history of hypertension (*p* < 0.000) and work ability index.

## 4 DISCUSSION

This study examined the correlation between WA, anthropometric indices, and cardiovascular risk factors among PSE. Findings showed that 57.5% were at least overweight, 38.6% had a history of hypertension, and 67.7% had never taken part in any physical activity, which reflects sedentary living (Table 3). This finding reflects the increase in the current burdens of obesity and hypertension in the working class or labor force.^50^, ^51^ The lifestyle issues also reflect the contribute of Saeedi et al's.^52^, ^53^ who predicted 10.2% increase in diabetes by 2030 and 10.9% by 2045.

Findings showed that 51.2% of the study population had good, 3.9% had moderate, and 44.9% had excellent WA (Table 1), indicating a highly competent population. This suggests that, in terms of work demands, health, and mental resources, the participants can currently perform their tasks and, in the future, increase productivity and the socioeconomic growth of the country, which is in line with Walker & Maltby.^54^ previous submission that highly skilled workers labored longer.

This study demonstrated a substantial link between history of diabetes, hypertension, alcohol consumption, smoking, or physical activity, and work performance (Table 6), which support earlier literature that lifestyle decisions can cause CVD and its clinical appearance.^55^ Poor diet, inactivity, smoking, and dangerous drinking are the main ones. Since most participants in this study had no comorbidities, behavioral risk factors alone cannot fully explain the prevalence of CVDs in this population.

This study demonstrated a strong correlation between physiological factors, including increased systolic BP, and anthropometric indices like weight, BMI, WC, weight‐to‐hip ratio, weight‐to‐height ratio, and work capacity as seen in literature.^56^, ^57^, ^58^ This study found that demographic and lifestyle characteristics of age, marital status, BMI, and number of children correlated with job performance as recently submitted by Kaewdok et al.^20^ that a person's physical attributes like age, weight, and musculoskeletal capabilities and lifestyle variables like leisure‐time physical activity, diet, smoking, and sleep and his job capacity index.

### 4.1 Conclusion

The majority of participants had at least moderate WA, which is associated with a prolonged active work life. Nonetheless, there was evidence of correlation between hypertension, physical inactivity, overweight and obesity, increasing WC, and increasing waist‐to‐hip ratio among participants whose work schedules left them with little or no time for healthy living, as well as the association between WA and anthropometric indices and CVDs risk factor components. To reduce the burden of CVDs in Ghana, early detection and control of risk factors, prevention and education programs, treatment, control of noncommunicable diseases, and lifestyle modifications must be intensified to enhance the health profile and quality of life.

### 4.2 Recommendation

Cardiovascular disease can be prevented by limiting behavioral risk factors such as cigarette use, poor diet, obesity, physical inactivity, and problematic alcohol consumption. To reduce CVDs risk factors and enhance the health profile and quality of life, it is necessary to intensify lifestyle modification education. Workplace health care strategy should be put in place to control BP, BMI, WC, WHR, weight to height ratio, and visceral fat as CVDs risk factors.

## AUTHOR CONTRIBUTIONS

**Angela Arthur**: Conceptualization; formal analysis; investigation; methodology; project administration; writing—original draft; writing—review and editing. **Anthony Mensah‐Asamoah**: Conceptualization; methodology; project administration; supervision; writing—original draft; writing—review and editing. **Eleazer Kofi Mensah Brown**: Formal analysis; methodology; writing—original draft; writing—review and editing. **Anita Kabuki Ocansey**: Formal analysis; methodology; project administration; writing—original draft; writing—review and editing. **Prince De‐Gaulle Deku**: Formal analysis; methodology; project administration; writing—original draft; writing—review and editing. **Monday Omoniyi Moses**: Conceptualization; formal analysis; methodology; project administration; resources; supervision; writing—original draft; writing—review and editing.

## CONFLICT OF INTEREST STATEMENT

The author declare no conflict of interest.

## TRANSPARENCY STATEMENT

The lead author Monday Omoniyi Moses affirms that this manuscript is an honest, accurate, and transparent account of the study being reported; that no important aspects of the study have been omitted; and that any discrepancies from the study as planned (and, if relevant, registered) have been explained.

## Data Availability

The data that support the findings of this study are available on request from the corresponding author (*). The data are not publicly available due to restrictions associated with participants' information that could compromise the privacy of research participants. All authors have read and approved the final version of the manuscript. The corresponding author had full access to all the data in this study and takes complete responsibility for the integrity of the data and the accuracy of the data analysis.
